# Prevalence of overweight, obesity and metabolic syndrome in HIV-infected patients

**DOI:** 10.1186/1758-5996-7-S1-A98

**Published:** 2015-11-11

**Authors:** Rebeca Antunes Beraldo, Giovanna Soler Donofre, Anderson Maliere Navarro, Valdes Roberto Bollela, Andre Schmidt, Maria Cristina Foss-Freitas

**Affiliations:** 1 Faculdade de Medicina de Ribeirão Preto – USP, Ribeirão Preto, Brazil

## Background

Lipodystrophy syndrome or HIV metabolic syndrome is characterized by alterations in the lipid and glucose metabolism, excess and redistribution of body fat and hypertension. Despite the highly active antiretroviral therapy (HAART) bring many benefits to carriers of the HIV virus, metabolic changes can occur as side effects, increasing cardiovascular risks.

## Objective

To assess the prevalence of overweight, obesity and metabolic syndrome in HIV-infected patients on HAART.

## Materials and methods

We measured the weight and height and calculated body mass index (BMI). We also performed biochemical tests of lipid profile and fasting glucose. Systemic blood pressure was measured on the right side using a digital sphygmomanometer. Waist circumference was measured from the midpoint between the last rib and the iliac crest. The criteria proposed by the National Cholesterol Education Program III (NCEP-ATP III) to metabolic syndrome classification were used.

## Results

We studied 281 patients (120 female and 161 male) with a mean age of 44.0 (±10.2) yrs. BMI averaged 25.82 (±5.65) kg/m^2^. The prevalence of obesity was 18.50% and the overweight, 31.31%. Metabolic syndrome was present in 51.24% of patients (55.00% in females and 48.4% in males).

## Conclusions

The high prevalence of overweight/obesity and metabolic syndrome highlights the importance of early nutritional intervention to prevent cardiovascular complications in this group of patients.

